# Identification of fatty acid metabolism hub genes in endometriosis using integrative bioinformatics analysis

**DOI:** 10.3389/fmed.2025.1529074

**Published:** 2025-04-17

**Authors:** Jiang-Lie Tu, Rui-Xue Fang

**Affiliations:** ^1^Department of Obstetrics and Gynecology, The Affiliated Hospital of Guizhou Medical University, Guizhou Hospital of The First Affiliated Hospital, Sun Yat-sen University, Guiyang, Guizhou, China; ^2^Department of Emergency, Yueqing Fifth People’s Hospital, Wenzhou, Zhejiang, China

**Keywords:** endometriosis, fatty acid metabolism, bioinformatic analysis, hub genes, PPI

## Abstract

**Background:**

Fatty acid metabolism plays a major role in several inflammatory diseases such as endometriosis. However, its specific mechanism in endometriosis remains unclear. Therefore, this study aimed to investigate the hub genes involved in endometriosis and fatty acid metabolism using bioinformatics analyses.

**Methods:**

The R package sva was used to remove batch effects from the GSE120103 and GSE25628 datasets, resulting in the creation of a combined GEO dataset. Differential analysis of the combined GEO dataset was interposed with fatty acid metabolism-related genes. Differentially expressed genes associated with fatty acid metabolism (FAMRDEGs) were subsequently identified. Functional enrichment analyses were performed using the clusterProfiler package, whereas gene set enrichment analysis (GSEA) was used to identify significant pathways. Protein–protein interaction (PPI) networks were constructed using STRING and visualized using Cytoscape to identify hub genes. Moreover, regulatory networks involving transcription factors and microRNAs were constructed using ChIPBase and ENCORI databases, respectively. Hub genes were validated via expression comparison and receiver operating characteristic curve analysis.

**Results:**

We identified 405 DEGs in the combined dataset, including 168 and 237 with upregulated and downregulated expression, respectively. Of these, 17 were FAMRDEGs. These genes were significantly involved in arachidonic acid and fatty acid metabolic processes. GSEA highlighted pathways such as Hamai_apoptosis_via_trail_dn for genes whose expression was downregulated, along with nuclear receptors in lipid metabolism and toxicity for genes with upregulated expression. The PPI network identified six hub genes: *PTGS2, CYP2C9, HSDL2, HSD17B3, ACSL4*, and *CYP2C18*. *ACSL4* showed the strongest positive correlation with immune cell effector memory CD8 T cells, whereas *HSDL2* showed the strongest negative correlation with immune cell-activated CD8 T cells.

**Conclusion:**

The identified hub genes may be potential biomarkers of fatty acid metabolism in endometriosis. This reveals the potential molecular mechanisms underlying this metabolic process and identifies therapeutic targets for future interventions.

## 1 Introduction

Endometriosis is an estrogen-dependent chronic inflammatory disease (1). According to the World Health Organization, approximately 10% of women of reproductive age are diagnosed with this condition worldwide (2). Despite the high prevalence of endometriosis, its pathogenesis remains unclear; this complicates both diagnosis and treatment. Current therapeutic approaches, including hormonal therapy and surgical interventions, often provide temporary relief. In addition, they often have side effects and are associated with a notably high recurrence rate post-surgery (3). Because the symptoms associated with endometriosis are frequently misattributed to dysmenorrhea, a condition commonly experienced by adolescent girls and young women, significant delays in diagnosis can occur (4). Therefore, further studies are required to better understand the underlying pathological mechanisms and develop new diagnostic and therapeutic strategies. Recent investigations have indicated that endometriosis should not be viewed solely as a localized condition; rather, it is associated with systemic alterations, including modifications in lipid metabolism.

Fatty acid metabolism plays an essential role in the pathophysiology of various inflammatory disorders (5). Notable alterations have been observed in the lipid profiles of women diagnosed with endometriosis, indicating a potential correlation between lipid metabolism and disease progression (6). In addition, genes related to A are involved in the regulation of inflammatory responses, which are pivotal for the development and maintenance of endometriotic lesions (7). These observations suggest that fatty acid metabolism-related genes (FAMRGs) are intricately associated with the onset and progression of endometriosis. However, a systematic investigation into the hub genes and potential regulatory mechanisms associated with fatty acid metabolism in the context of endometriosis remains to be conducted. Therefore, this study aimed to evaluate the differential expression of FAMRGs in endometriosis and explore the potential regulatory mechanisms involved.

Our findings highlight the potential application of fatty acid metabolism hub genes as diagnostic markers and therapeutic targets in endometriosis. This study further elucidates the molecular mechanisms underlying the pathogenesis of this disease and may provide valuable insights for developing new diagnostic markers and therapeutic targets.

## 2 Materials and methods

### 2.1 Data used

The endometriosis datasets GSE120103 and GSE25628 were downloaded from the GEO database^1^ using the R package GEO query (Version 2.72.0). These datasets were extracted from human endometrial tissues. The chip platforms of GSE120103 and GSE25628 were GPL6480 and GPL571, respectively (Table 1). In total, 849 genes related to fatty acid metabolism were identified based on previous literature (8–10) after combination and deduplication (Supplementary Table S1).

**Table 1:** GEO microarray chip information.

	GSE120103	GSE25628
Platform	GPL6480	GPL571
Species	*Homo sapiens*	*Homo sapiens*
Tissue	Endometriosis tissues	Endometriosis tissues
Samples in EMs group	18	16
Samples in control group	18	6
Reference	PMID: 30760267	PMID: 23460397

GEO, Gene Expression Omnibus; EMs, Endometriosis.

### 2.2 Data preprocessing

The R package sva (version 3.52.0) was used to remove batch effects from the two datasets, resulting in the creation of a combined GEO dataset. The combined dataset included 33 endometriosis and 25 control samples. Finally, the R package limma (version 3.60.2) was used to standardize and normalize the integrated GEO dataset and annotate probes. Principal component analysis (PCA) was performed on the expression matrix, both before and after the removal of the batch effect, to assess the effectiveness of batch effect removal.

### 2.3 Identification of endometriosis-associated fatty acid metabolism-related differentially expressed genes

The data were divided into the Endometriosis and Control groups. The R package limma (version 3.60.2) was used to perform differential analysis of genes in the two groups. Genes with threshold values of |logFC| > 1 and *p* < 0.05 were considered differentially expressed genes (DEGs). The R package ggplot2 (version 3.5.1) was used to plot the results of the differential analysis as volcano plots. The intersection of DEGs and FAMRGs was subsequently determined, and a Venn diagram was drawn to obtain FAMRDEGs. The R packages pheatmap (version 1.0.12) and RCircos (version 1.2.2) were used to draw a heatmap and a chromosome localization map, respectively.

### 2.4 Functional enrichment analysis

The R package clusterProfiler (version 4.12.0) was used to perform gene ontology (GO) and pathway (KEGG) enrichment analyses on FAMRDEGs. The entry screening criteria were adj. *p* < 0.05 and *q* < 0.25.

### 2.5 Gene set enrichment analysis (GSEA)

Genes in the combined GEO datasets were sorted according to their logFC values. GSEA was performed using the R package clusterProfiler (version 4.12.0) for all genes in the combined datasets. The c2 gene set was obtained using the R package msigdbr (version 7.5.1) before GSEA was performed. The screening criteria for GSEA were adj. *p* < 0.05 and *q* < 0.25.

### 2.6 Protein–protein interaction (PPI) network

The STRING database^2^ was applied based on FAMRDEGs with a minimum interaction coefficient >0.4 to construct a PPI network related to these genes. The Cytoscape software was used to visualize the networks. The MCC algorithm in the CytoHubba plug-in of Cytoscape was used to calculate the scores of FAMRDEGs. Next, the top six FAMRDEGs were selected as related hub genes based on their scores. We predicted functionally similar hub genes using the GeneMANIA database^3^ to construct a PPI network.

### 2.7 Construction of regulatory network

The ChIPBase database^4^ was used to retrieve transcription factors (TFs). The regulatory function of TFs in hub genes was analyzed, and the mRNA-TF regulatory network was visualized using Cytoscape software. Hub genes associated with microRNAs (miRNAs) were retrieved from the ENCORI database^5^ to evaluate the relationship between hub genes and miRNA. The mRNA-miRNA regulatory network was subsequently visualized using Cytoscape software.

### 2.8 Differential expression verification and receiver operating characteristic (ROC) curve analysis of hub genes

A comparative chart was constructed based on the expression levels of hub genes to further investigate the differential expression of the hub genes between the Endometriosis and Control groups within the integrated GEO datasets. Next, the R package pROC (version 1.18.5) was used to generate an ROC curve for the hub genes, thereby enabling the calculation of the area under the curve (AUC). This analysis assessed the diagnostic efficacy of hub gene expression in relation to the occurrence of endometriosis.

### 2.9 Single-sample GSEA (ssGSEA)

ssGSEA was used to quantify the relative abundance of each immune cell type. We then used the R package ggplot2 (version 3.5.0) to create comparative visualizations that depicted expression differences in immune cells between the Control and H groups within the combined GEO dataset. Immune cells that demonstrated significant differences between the two groups were selected for further analysis. We applied the Spearman correlation algorithm to assess the correlation between immune cell types. The R package pheatmap (version 1.0.12) was used to create a correlation heatmap. The correlation between hub genes and immune cells was subsequently calculated using Spearman’s algorithm, and the results were retained at a *p*-value of <0.05. The R package ggplot2 (version 3.5.1) was used to draw a correlation bubble plot to show the correlation between hub genes and immune cells. Immune cells with TOP1-positive and TOP1-negative correlation with hub genes were identified, and a correlation scatter plot was drawn using ggplot2.

### 2.10 Statistical analyses

Statistical analyses were performed using the R statistical package (version 4.4.0; R Foundation for Statistical Computing, Vienna, Austria). The *p*-values were two-sided, and a p-value of <0.05 was considered statistically significant.

## 3 Results

### 3.1 Technology roadmap (Figure 1)

**Figure 1:**
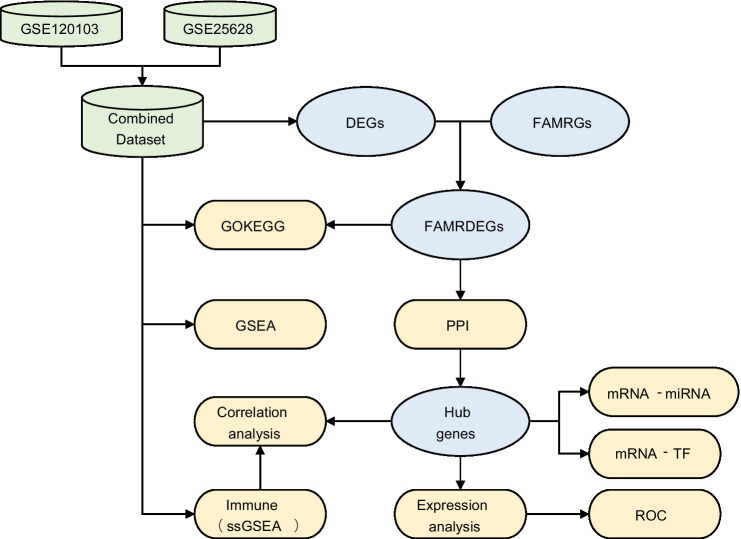
Flowchart for the comprehensive analysis of FAMRDEGs. EMs, endometriosis; DEGs, differentially expressed genes; FAMRGs, fatty acid metabolism-related genes; FAMRDEGs, fatty acid metabolism-related differentially expressed genes; GO, Gene Ontology; KEGG, Kyoto Encyclopedia of Genes and Genomes; GSEA, gene set enrichment analysis; PPI, protein–protein interaction; miRNA, MicroRNA; TF, transcription factor; ROC, receiver operating characteristic; ssGSEA, single-sample gene set enrichment analysis.

A flowchart illustrating the FAMRDEG analysis is shown in Figure 1.

### 3.2 Merging of endometriosis datasets

**Figure 2:**
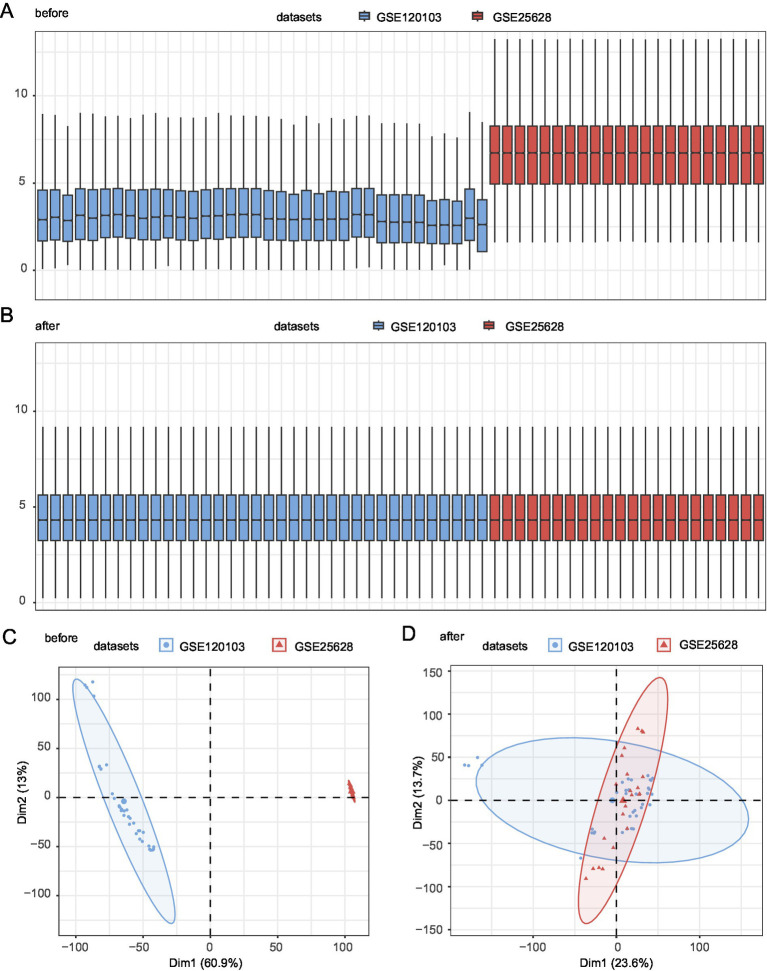
Batch effect removal for GSE73461 and GSE68004. **(A)** Boxplots showing the distribution of combined GEO datasets before batch removal. **(B)** Boxplots showing the distribution of post-batch integrated GEO datasets (combined datasets). **(C)** The PCA dataset before batch processing. **(D)** The PCA map of the combined GEO datasets after batch processing. Light red and blue represent the EMs datasets GSE25628 and GSE120103, respectively. PCA, principal component analysis; EMs, endometriosis.

The endometriosis datasets GSE120103 and GSE25628 were processed using the R package sva to remove the batch effect and obtain a combined GEO dataset. The datasets before and after batch effects were compared using distribution box plots (Figures 2A,B) and PCA (Figures 2C,D). The batch effect in the dataset was successfully eliminated after batch processing.

### 3.3 DEGs related to endometriosis-associated fatty acid metabolism

**Figure 3:**
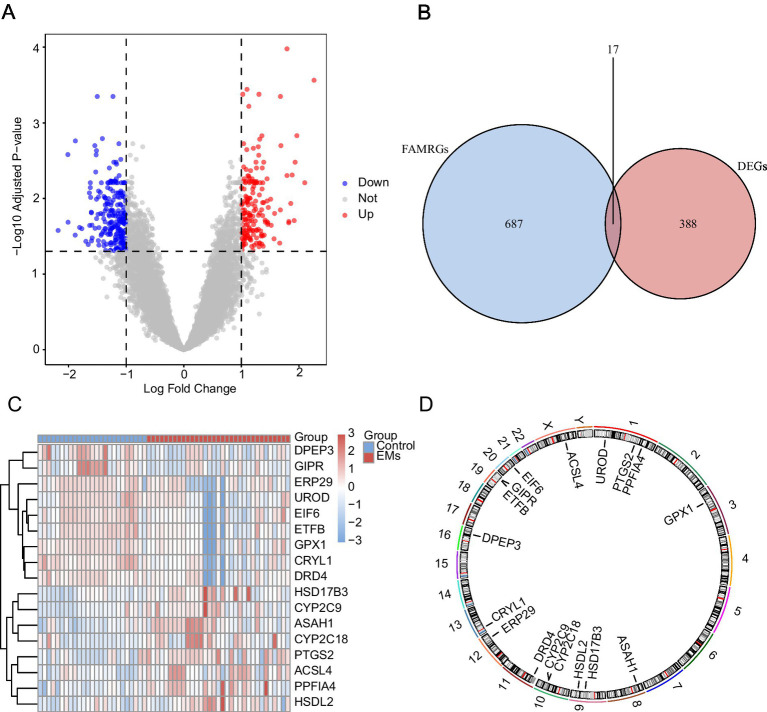
Differential gene expression analysis. **(A)** Volcano plot of DEG analysis between the EMs and control groups in the combined GEO datasets. **(B)** A Venn diagram of DEGs and FAMRGs in the integrated GEO datasets (combined datasets). **(C)** A heatmap of FAMRDEGs in the integrated GEO datasets (combined datasets). **(D)** Chromosomal mapping of FAMRDEGs. Light red and blue represent the EMs and Control groups, respectively. In the heatmap, red and blue represent high and low expressions, respectively. EMs, endometriosis; DEGs, differentially expressed genes; FAMRGs, fatty acid metabolism-related genes; FAMRDEGs, fatty acid metabolism-related differentially expressed genes.

The R package limma identified 405 DEGs in the combined dataset. Of these, the expression of 168 genes was upregulated, whereas that of 237 genes was downregulated. A volcano diagram for this dataset was drawn based on the results of differential analysis (Figure 3A). Moreover, a Venn diagram of all DEGs and FAMRGs was drawn to obtain FAMRDEGs (Figure 3B). In total, 17 FAMRDEGs were obtained: *CRYL1, ASAH1, HSD17B3, DPEP3, PTGS2, PPFIA4, EIF6, DRD4, UROD, ETFB, CYP2C18, GIPR, CYP2C9, ACSL4, ERP29, HSDL2,* and *GPX1*. Differences in the expression of FAMRDEGs between sample groups in the combined GEO datasets were analyzed based on the intersection results. A heatmap of the analysis results is shown in Figure 3C. In addition, the chromosome localization map of the 17 FAMRDEGs is shown in Figure 3D. Chromosome mapping showed that FAMRDEGs were mostly located on chromosomes 1, 9, 10, and 19. *UROD, PTGS2,* and *PPFIA4* were located on chromosome 1; *HSDL2* and *HSD17B3* were located on chromosome 9; *CYP2C9* and *CYP2C18* were located on chromosome 10; and *GIPR* and *ETFB* were located on chromosome 19.

### 3.4 Functional enrichment analysis

**Figure 4:**
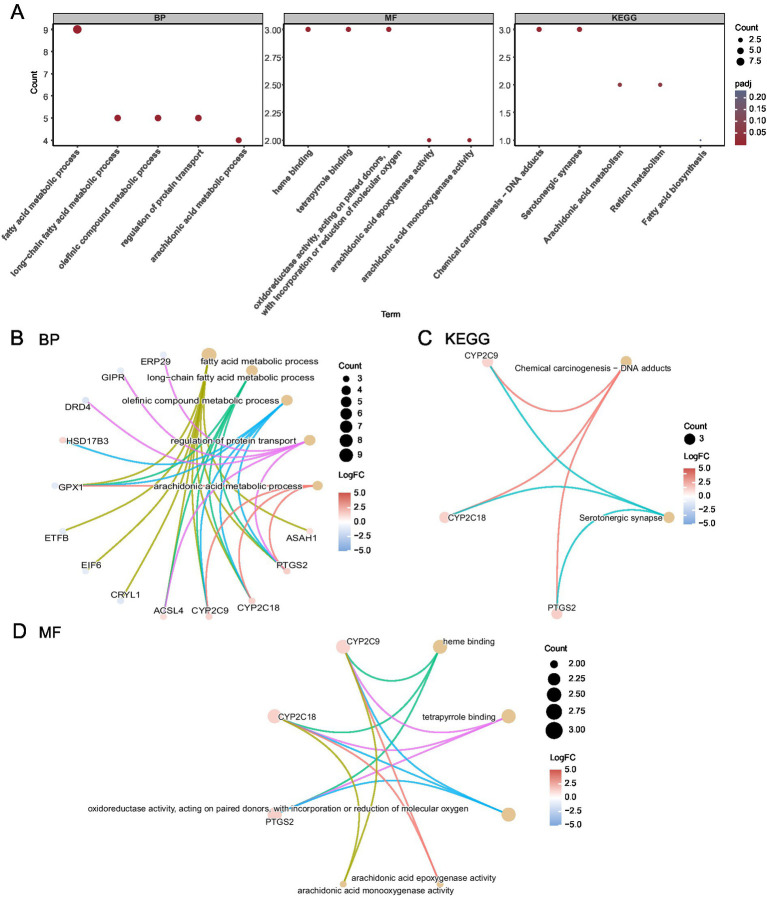
GO and KEGG enrichment analyses for FAMRDEGs. **(A)** A bubble plot of GO and KEGG enrichment analysis results for BP, MF, and biological pathway (KEGG) in FAMRDEG. GO and KEGG terms are shown on the abscissa. **(B–D)** GO and KEGG enrichment analysis results for the network diagram of FAMRDEGs showing BP **(B)**, KEGG **(C)**, and MF **(D)**. Lighter red and light brown nodes represent items, and nodes represent molecules. The attachment shows the genes and their corresponding access nodes. Larger pathway nodes indicate greater enrichment of the pathways in genes. The color of the gene node indicates its logFC. Red and blue represent upregulated and downregulated gene expression, respectively. The bubble size and color represent the number of genes and the size of the adj. *p*-value, respectively. A deeper red color indicates a smaller adj. *p*-value, whereas a bluer color indicates a larger adj. *p*-value. The screening criteria for GO and KEGG enrichment analyses were adj. *p* < 0.05 and FDR value *q* < 0.25, and the Benjamini–Hochberg *p*-value correction method was used. FAMRDEGs, fatty acid metabolism-related differentially expressed genes; GO, Gene Ontology; KEGG, Kyoto Encyclopedia of Genes and Genomes; BP, biological process; MF, molecular function.

The following GO and KEGG enrichment pathways were explored for the 17 FAMRGs: biological process (BP), cell component (CC), molecular function (MF), biological pathways (KEGG), and their relationship to endometriosis (EMs). The 17 FAMRDEGs used for GO and KEGG enrichment analyses are listed in Table 2. These genes were primarily enriched in arachidonic acid and fatty acid metabolic processes related to endometriosis. However, they were not enriched in the following pathways: BP, such as long-chain fatty acid and olefinic compound metabolic processes, regulation of protein transport, arachidonic acid epoxygenase activity, heme binding, oxidoreductase activity, acting on paired donors, and incorporation or reduction of molecular oxygen; MF, such as arachidonic acid monooxygenase and tetrapyrrole binding; or CC. In contrast, these genes were enriched in KEGG pathways including chemical carcinogenesis, DNA adducts, and serotonergic synapse. The results of the GO and KEGG enrichment analyses were visualized using bubble plots (Figure 4A). The network diagrams for BP, MF, and KEGG are shown in Figures 4B–D.

**Table 2:** Results of GO and KEGG enrichment analysis for FAMRDEGs.

Ontology	ID	Description	Gene ratio	Bg ratio	*p* value	p adj	*q* value
BP	GO:0006631	fatty acid metabolic process	9/17	401/18888	1.68E-11	1.22E-08	6.97E-09
BP	GO:0001676	long-chain fatty acid metabolic process	5/17	109/18888	3.42E-08	1.24E-05	7.10E-06
BP	GO:0120254	olefinic compound metabolic process	5/17	162/18888	2.48E-07	4.52E-05	2.58E-05
BP	GO:0051223	regulation of protein transport	5/17	440/18888	3.29E-05	0.003424	0.001956
BP	GO:0019369	arachidonic acid metabolic process	4/17	58/18888	1.85E-07	4.49E-05	2.56E-05
MF	GO:0020037	heme binding	3/17	142/18522	0.000277	0.004808	0.001568
MF	GO:0046906	tetrapyrrole binding	3/17	152/18522	0.000339	0.004808	0.001568
MF	GO:0016705	oxidoreductase activity, acting on paired donors, with incorporation or reduction of molecular oxygen	3/17	179/18522	0.000546	0.006464	0.002108
MF	GO:0008392	arachidonic acid epoxygenase activity	2/17	16/18522	9.44E-05	0.00427	0.001393
MF	GO:0008391	arachidonic acid monooxygenase activity	2/17	18/18522	0.00012	0.00427	0.001393
KEGG	hsa05204	Chemical carcinogenesis – DNA adducts	3/13	71/8840	0.000134	0.006702	0.006209
KEGG	hsa04726	Serotonergic synapse	3/13	115/8840	0.000558	0.013944	0.012917
KEGG	hsa00590	Arachidonic acid metabolism	2/13	61/8840	0.003479	0.053821	0.049856
KEGG	hsa00830	Retinol metabolism	2/13	68/8840	0.004306	0.053821	0.049856
KEGG	hsa00061	Fatty acid biosynthesis	1/13	18/8840	0.026167	0.22962	0.212701

GO, Gene Ontology; BP, Biological Process; CC, Cellular Component; MF, Molecular Function; KEGG, Kyoto Encyclopedia of Genes and Genomes; FAMRDEGs, Fatty Acid Metabolism-Related Differentially Expressed Genes.

### 3.5 GSEA

**Figure 5:**
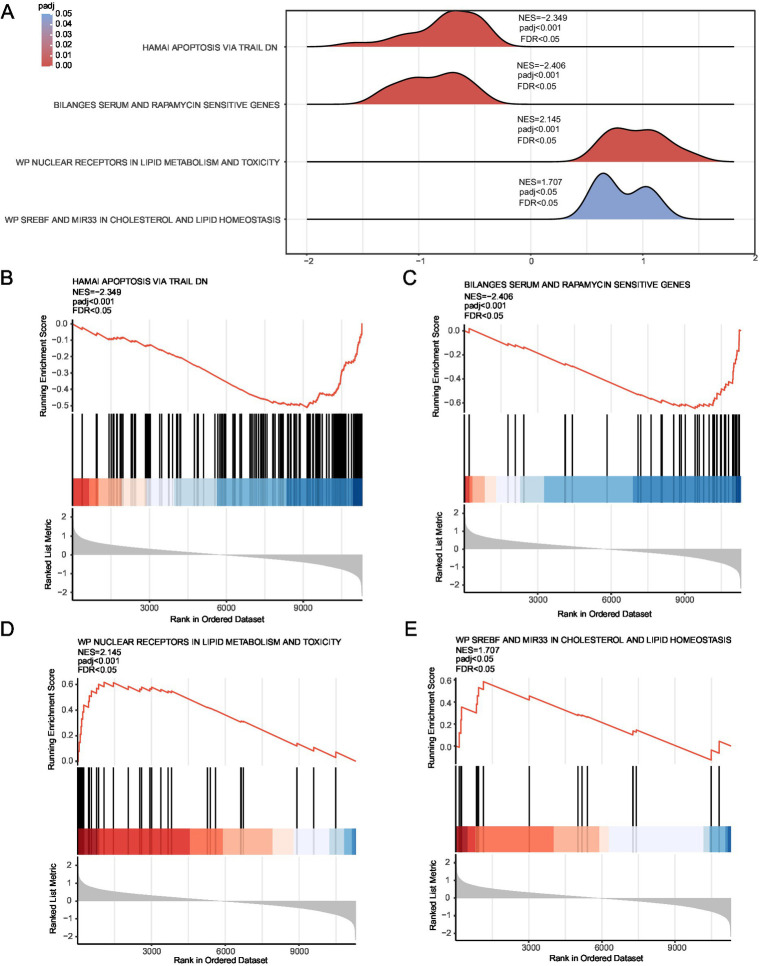
GSEA for the combined dataset. **(A)** Mountain map display of four biological functions from the GSEA of combined GEO datasets. **(B–E)** GSEA results for genes with downregulated **(B,C)** and upregulated **(D,E)** expression. The screening criteria for GSEA were adj. *p* < 0.05 and FDR value *q* < 0.25, and the Benjamini–Hochberg *p*-value correction method was used. GSEA, gene set enrichment analysis.

The effect of all gene expression levels in the combined GEO datasets was evaluated. The GSEA results for genes involved in BP, CC, and MF are shown in Figure 5A and Table 3. Genes whose expression was downregulated in the combined datasets were significantly enriched in hamai apoptosis via TRAIL DN (Figure 5B), bilanges serum, as well as rapamycin sensitivity (Figure 5C) and other biologically relevant functions and signaling pathways. In contrast, genes whose expression was upregulated were significantly enriched in nuclear receptors in lipid metabolism and toxicity (Figure 5D), as well as srebf and mir33 in cholesterol and lipid homeostasis (Figure 5E) and other biologically related functions and signaling pathways.

**Table 3:** Results of GSEA for combined datasets.

ID	Set size	Enrichment score	NES	*p* value	*p* adjust	*q* value
HSIAO HOUSEKEEPING GENES	326	−0.64948	−3.26751	1.00E-10	9.95E-09	7.43E-09
REACTOME EUKARYOTIC TRANSLATION ELONGATION	68	−0.77561	−3.16842	1.00E-10	9.95E-09	7.43E-09
REACTOME EUKARYOTIC TRANSLATION INITIATION	93	−0.72453	−3.14164	1.00E-10	9.95E-09	7.43E-09
WP CYTOPLASMIC RIBOSOMAL PROTEINS	67	−0.76639	−3.109	1.00E-10	9.95E-09	7.43E-09
KEGG RIBOSOME	65	−0.76707	−3.09318	1.00E-10	9.95E-09	7.43E-09
REACTOME RESPONSE OF EIF2AK4 GCN2 TO AMINO ACID DEFICIENCY	77	−0.73949	−3.0839	1.00E-10	9.95E-09	7.43E-09
REACTOME NONSENSE MEDIATED DECAY NMD	89	−0.70339	−3.02811	1.00E-10	9.95E-09	7.43E-09
REACTOME SRP DEPENDENT COTRANSLATIONAL PROTEIN TARGETING TO MEMBRANE	86	−0.70743	−3.00935	1.00E-10	9.95E-09	7.43E-09
REACTOME SELENOAMINO ACID METABOLISM	81	−0.712	−2.9979	1.00E-10	9.95E-09	7.43E-09
REACTOME INFLUENZA INFECTION	125	−0.65741	−2.97902	1.00E-10	9.95E-09	7.43E-09
REACTOME REGULATION OF EXPRESSION OF SLITS AND ROBOS	134	−0.63804	−2.91137	1.00E-10	9.95E-09	7.43E-09
REACTOME CELLULAR RESPONSE TO STARVATION	113	−0.65122	−2.89995	1.00E-10	9.95E-09	7.43E-09
REACTOME TRANSLATION	197	−0.60453	−2.89103	1.00E-10	9.95E-09	7.43E-09
REACTOME RRNA PROCESSING	145	−0.61885	−2.84159	1.00E-10	9.95E-09	7.43E-09
KIM ALL DISORDERS DURATION CORR DN	127	−0.61318	−2.78356	1.00E-10	9.95E-09	7.43E-09
PECE MAMMARY STEM CELL UP	98	−0.63343	−2.77538	1.00E-10	9.95E-09	7.43E-09
CHNG MULTIPLE MYELOMA HYPERPLOID UP	38	−0.77545	−2.7727	1.00E-10	9.95E-09	7.43E-09
REACTOME ACTIVATION OF THE MRNA UPON BINDING OF THE CAP BINDING COMPLEX AND EIFS AND SUBSEQUENT BINDING TO 43S	47	−0.72904	−2.74679	1.00E-10	9.95E-09	7.43E-09
REACTOME SIGNALING BY ROBO RECEPTORS	175	−0.5808	−2.73332	1.00E-10	9.95E-09	7.43E-09
YAO TEMPORAL RESPONSE TO PROGESTERONE CLUSTER 17	145	−0.58658	−2.69339	1.00E-10	9.95E-09	7.43E-09

GSEA, Gene Set Enrichment Analysis; NES, Controlized Enrichment Score.

### 3.6 Construction of the PPI network and screening of hub genes

**Figure 6:**
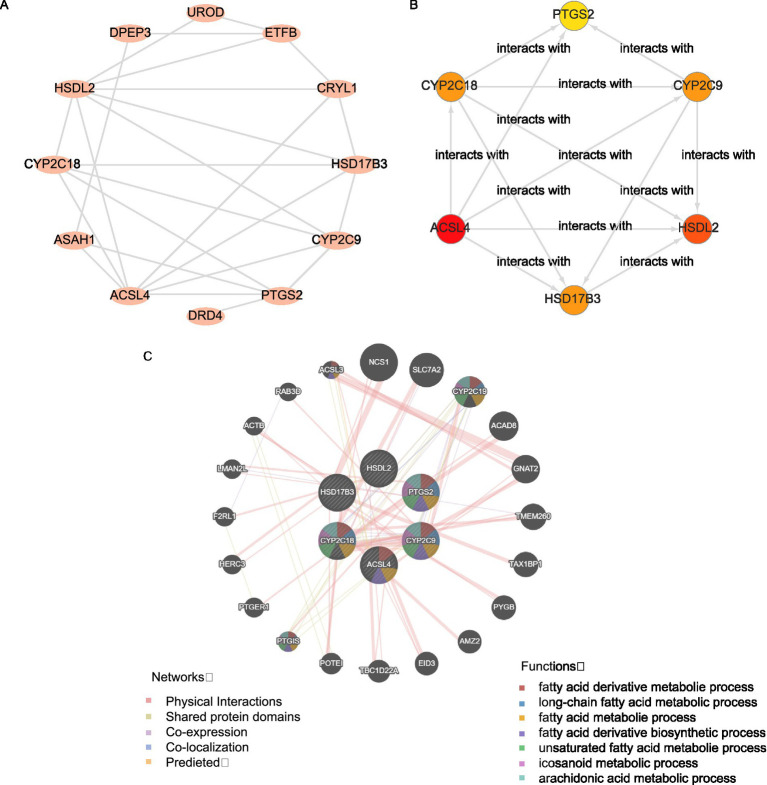
Interaction network analysis for key genes. **(A)** PPI network of FAMRDEGs, as calculated using the STRING database. **(B)** The PPI network of the top six FAMRDEGs (*PTGS2, CYP2C9, HSDL2 HSD17B3, ACSL4,* and *CYP2C18*). The circle color (from red to yellow) represents the score (from high to low). **(C)** GeneMANIA forecasts the interaction network for hub genes and other genes of similar functions. Lines with different colors represent co-expression and shared information, such as protein domains. The Hub circle represents the hub genes identified in the network, while the colored attachments indicate genes with similar functions. Corresponding to the color of each connection reflects different types of functional relationships, such as co-expression or shared protein domains.

The PPI network of 17 FAMRDEGs is shown in Figure 6A. In total, 12 FAMRDEGs were related: *UROD, ETFB, CRYL1, HSD17B3, CYP2C9, PTGS2, DRD4, ACSL4, ASAH1, CYP2C18, HSDL2,* and *DPEP3*. The scores of these genes were subsequently calculated, and the top six genes were screened based on these scores. The interaction network is shown in Figure 6B. Six hub genes were identified, namely, *PTGS2, CYP2C9, HSDL2, HSD17B3, ACSL4,* and *CYP2C18*.

Finally, the GeneMANIA website predicted an interaction network between the six hub genes and genes with similar functions (Figure 6C). Six hub genes and 20 functionally similar proteins were identified.

### 3.7 Construction of regulatory network

**Figure 7:**
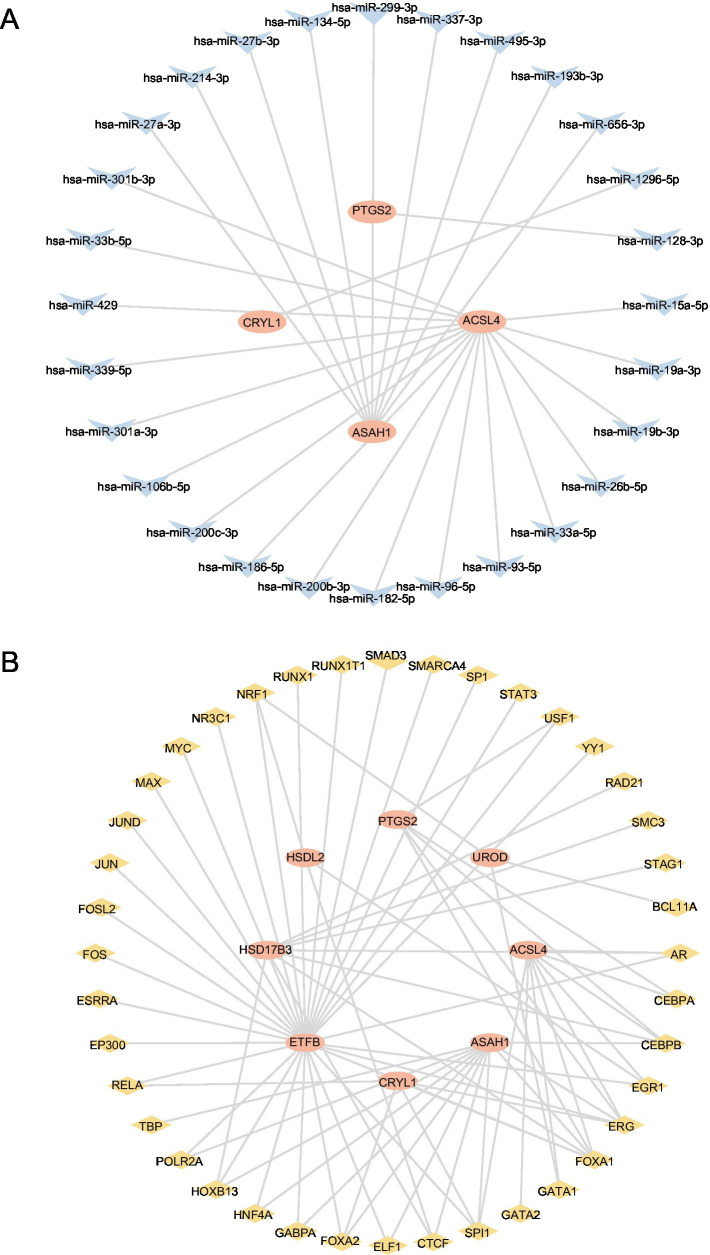
Construction of the regulatory network of FAMRDEGs. The mRNA-miRNA **(A)** and mRNA-TF **(B)** regulatory networks of FAMRDEGs. Orange, light blue, and pale yellow represent mRNA, miRNA, and TF, respectively. FAMRDEGs, fatty acid metabolism-related differentially expressed genes; miRNA, MicroRNA; TF, transcription factor.

We used the ENCORI database to obtain microRNAs associated with the hub genes *PTGS2, CYP2C9, HSDL2, HSD17B3, ACSL4,* and *CYP2C18*. The constructed mRNA-miRNA regulatory network is shown in Figure 7A. Four hub genes and 28 miRNAs were identified (Table 4).

**Table 4:** mRNA-miRNA interaction of key genes.

mRNA	miRNA
ACSL4	hsa-miR-15a-5p
ACSL4	hsa-miR-19a-3p
ACSL4	hsa-miR-19b-3p
ACSL4	hsa-miR-26b-5p
ACSL4	hsa-miR-33a-5p
ACSL4	hsa-miR-93-5p
ACSL4	hsa-miR-96-5p
ACSL4	hsa-miR-182-5p
ACSL4	hsa-miR-200b-3p
ACSL4	hsa-miR-186-5p
ACSL4	hsa-miR-200c-3p
ACSL4	hsa-miR-106b-5p
ACSL4	hsa-miR-301a-3p
ACSL4	hsa-miR-339-5p
ACSL4	hsa-miR-429
ACSL4	hsa-miR-33b-5p
ACSL4	hsa-miR-301b-3p
ASAH1	hsa-miR-27a-3p
ASAH1	hsa-miR-214-3p
ASAH1	hsa-miR-27b-3p
ASAH1	hsa-miR-134-5p
ASAH1	hsa-miR-299-3p
ASAH1	hsa-miR-337-3p
ASAH1	hsa-miR-495-3p
ASAH1	hsa-miR-193b-3p
ASAH1	hsa-miR-656-3p
CRYL1	hsa-miR-1296-5p
PTGS2	hsa-miR-128-3p

miRNA, MicroRNA.

The ChIPBase database was used to construct the regulatory network of TFs in hub genes. The mRNA-TF regulatory network is shown in Figure 7B. Eight hub genes and 40 TFs were identified in this regulatory network (Table 5).

**Table 5:** mRNA-TF interaction of key genes.

mRNA	TF
ACSL4	AR
ACSL4	CEBPA
ACSL4	CEBPB
ACSL4	EGR1
ACSL4	ERG
ACSL4	FOXA1
ACSL4	GATA1
ACSL4	GATA2
ACSL4	SPI1
ASAH1	CEBPB
ASAH1	CTCF
ASAH1	ELF1
ASAH1	ERG
ASAH1	FOXA1
ASAH1	FOXA2
ASAH1	GABPA
ASAH1	HNF4A
ASAH1	HOXB13
ASAH1	POLR2A
ASAH1	SPI1
ASAH1	TBP
CRYL1	SPI1
CRYL1	ERG
CRYL1	FOXA1
CRYL1	FOXA2
CRYL1	RELA
ETFB	AR
ETFB	EGR1
ETFB	ELF1
ETFB	EP300
ETFB	ERG
ETFB	ESRRA
ETFB	FOS
ETFB	FOSL2
ETFB	FOXA1
ETFB	FOXA2
ETFB	GABPA
ETFB	HNF4A
ETFB	HOXB13
ETFB	JUN
ETFB	JUND
ETFB	MAX
ETFB	MYC
ETFB	NR3C1
ETFB	NRF1
ETFB	POLR2A
ETFB	RELA
ETFB	RUNX1
ETFB	RUNX1T1
ETFB	SMAD3
ETFB	SMARCA4
ETFB	SP1
ETFB	SPI1
ETFB	STAT3
ETFB	USF1
ETFB	YY1
HSD17B3	AR
HSD17B3	CEBPB
HSD17B3	CTCF
HSD17B3	FOXA1
HSD17B3	HOXB13
HSD17B3	RAD21
HSD17B3	SMC3
HSD17B3	STAG1
HSDL2	CTCF
HSDL2	EGR1
HSDL2	NRF1
PTGS2	FOXA1
PTGS2	USF1
PTGS2	CEBPA
PTGS2	CEBPB
PTGS2	ERG
UROD	GATA1
UROD	NRF1
UROD	BCL11A

TF, Transcription factors.

### 3.8 Validation of differentially expressed hub genes and ROC curve analysis

**Figure 8:**
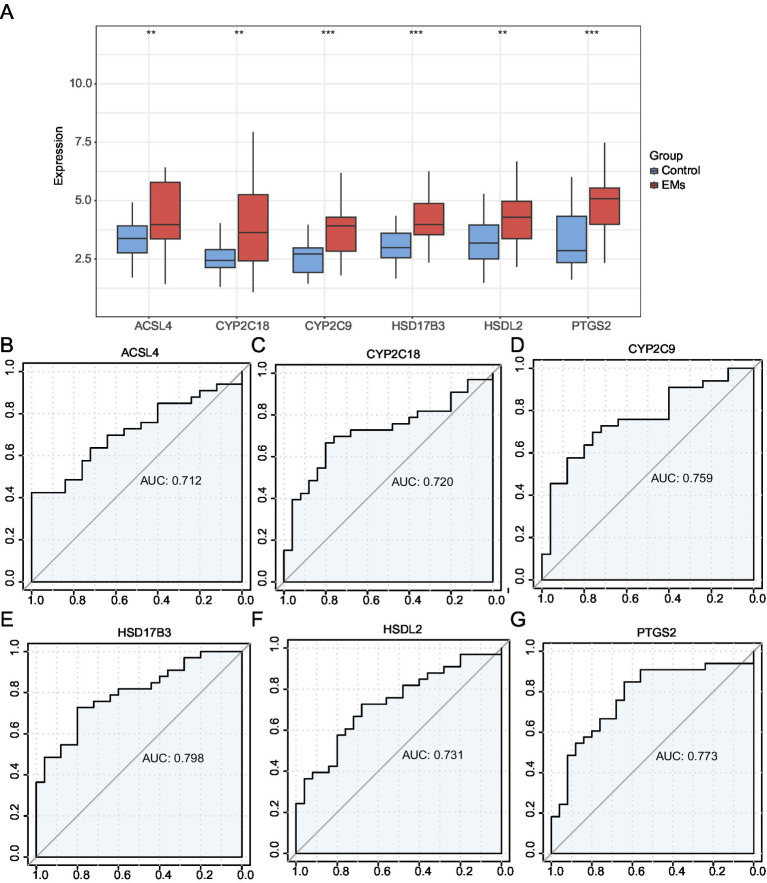
Validation of differential expression and ROC curve analysis. **(A)** Group comparison plot of hub genes in the EMs and control groups in the combined GEO datasets. **(B–G)** ROC curves of six hub genes in the integrated GEO Datasets: *ACSL4* **(B)**, *CYP2C18* **(C)**, *CYP2C9* **(D)**, *HSD17B3* **(E)**, *HSDL2* **(F)**, and *PTGS2* **(G)**. **p < 0.01, ***p < 0.001. EMs, endometriosis; ROC, receiver operating characteristic; AUC, area under the curve; TPR, true positive rate; FPR, false positive rate. Red and blue represent the Endometriosis and Control groups, respectively.

A grouping comparison chart was constructed to evaluate the differences in the expression of six hub genes between the Endometriosis and Control groups in the combined GEO datasets (Figure 8A). The analysis revealed statistically significant differences in the expression levels of high and low. Statistically significant differences were observed in the expression levels of six hub genes (*ACSL4, CYP2C18, CYP2C9, HSD17B3, HSDL2*, and *PTGS2*) between the Endometriosis and Control groups of the combined GEO datasets (*p* < 0.001). An ROC curve of expression levels of hub genes in the integrated GEO datasets is shown in Figures 8B–G. The expression levels of the six hub genes in the Endometriosis and Control groups were classified with high accuracy (0.7 < AUC < 0.9).

### 3.9 ssGSEA immune analysis

**Figure 9:**
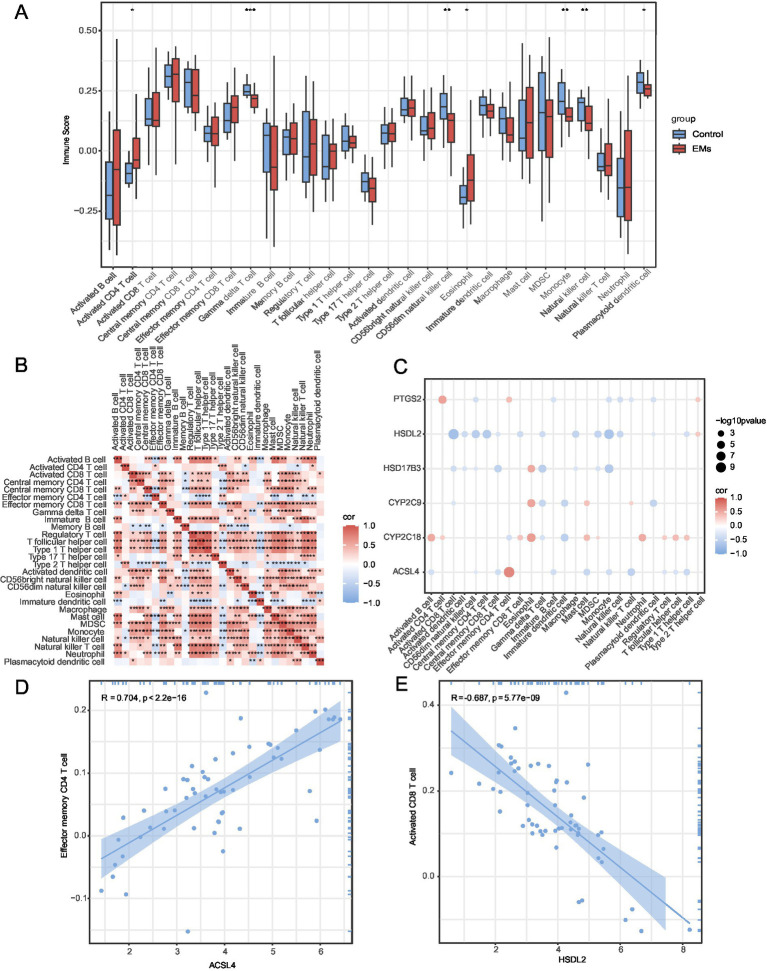
Immune infiltration analysis by ssGSEA algorithm. **(A)** Group comparison plot of immune cell infiltration in the Endometriosis and Control groups in combined GEO datasets. **(B)** A correlation heatmap of immune cell abundance. **(C)** A bubble plot showing the correlation between hub genes and immune cell infiltration in combined GEO datasets. **(D)** A scatter plot showing TOP1-positive correlation between hub genes and immune cells. **(E)** A scatter plot showing TOP1-negative correlation between hub genes and immune cells. ns represents *p* ≥ 0.05; *p < 0.05; **p < 0.01; and ***p < 0.001. *r* < 0.3, *r* = 0.3–0.5, *r* = 0.5–0.8, and *r* > 0.8 were considered weak or irrelevant, weak, moderate, and strong correlation, respectively. Light red and light blue represent the Endometriosis and Control groups, respectively. Red indicates a positive correlation, whereas blue indicates negative correlation. The depth of the color represents the strength of the correlation. ssGSEA, single-sample gene set enrichment analysis; EMs, endometriosis.

The abundance of 28 immune cell types was calculated using the ssGSEA algorithm. The group comparison diagram is shown in Figure 9A. Seven immune cells, namely, activated CD4 + T cells, gamma-delta T cells, CD56 dim natural killer cells, eosinophils, monocytes, natural killer T cells, and plasmacytoid dendritic cells, were significantly different between the Endometriosis and Control groups (*p* < 0.05). Correlation heatmaps of the immune infiltration of these cell types in the integrated GEO datasets are shown in Figure 9B. The association of six hub genes with these immune cells was analyzed using a correlation bubble chart (Figure 9C). The TOP1-positive and TOP1-negative correlation between hub genes and immune cells is shown in Figures 9D,E. *ACSL4* showed the strongest positive correlation with effector memory CD8 T cells (*r* = 0.704, *p* < 0.05) (Figure 9D), whereas *HSDL2* showed the strongest negative correlation with activated CD8 T cells (*r* = −0.687, *p* < 0.05) (Figure 9E).

## 4 Discussion

Endometriosis is a chronic gynecological disorder characterized by the presence of endometrial-like tissue outside the uterus; it results in inflammation, pain, and infertility (1). Despite the high prevalence of endometriosis, its pathogenesis remains unclear. This complicates both diagnosis and treatment. Current therapeutic approaches, including hormonal therapy and surgical interventions, often provide temporary relief. Furthermore, they have side effects and a notably high recurrence rate post-surgery (3). Because the symptoms associated with endometriosis are frequently misattributed to dysmenorrhea, a condition commonly experienced by adolescent girls and young women, significant delays in diagnosis can occur (4). This underscores the pressing need to elucidate the mechanisms underlying the pathogenesis of this disease and identify novel diagnostic markers. Endometriosis involves complex interactions between genetic, hormonal, and environmental factors (5). Recent studies have highlighted the role of metabolic dysregulation, particularly fatty acid metabolism, in the progression of this disease (7). However, the correlation between endometriosis and fatty acid metabolism remains largely understudied.

Endometriosis is a dynamic disease characterized by time-series changes in gene expression. Different stages of the disease involve distinct biological processes and molecular mechanisms, with fatty acid metabolism-related genes playing a key role (11). In the early stages, upregulation of fatty acid metabolism-related genes may help control local inflammatory responses and promote the repair of damaged tissue. For instance, these genes can increase the synthesis of anti-inflammatory substances and inhibit the production of pro-inflammatory factors, thereby reducing the inflammatory response. This mechanism is crucial for early response to disease progression. However, as the disease progresses, the expression of these genes may decline, leading to lipid metabolism imbalances and promoting the development of chronic inflammation and tissue remodeling.

Fluctuations in hormone levels significantly impact the regulation of fatty acid metabolism. In particular, estrogen and progesterone affect the synthesis, oxidation, and transportation of fatty acids. At different stages of the disease course, hormonal changes will directly affect the expression patterns of the genes, resulting in different clinical manifestations. For example, when hormone levels are elevated, the expression of related genes may promote an increase in fatty acid synthesis, which may be inhibited by a decrease in hormone levels. In addition, signaling pathways in cells are also closely related to changes in gene expression (12). For example, activation of inflammatory signaling pathways affects the expression of fatty acid metabolism-related genes, leading to changes in metabolites that may further promote disease progression. Together, these findings suggest that the pathological characteristics of endometriosis are closely linked to dynamic changes in gene expression, which may have distinct biological significance at different stages.

Seventeen FAMRDEGs were identified in the present study. These genes were significantly involved in arachidonic acid and fatty acid metabolic processes. GeneMANIA predicted a complex interaction network between six hub genes and 20 functionally similar proteins. The integration of gene expression data and functional analyses highlighted the potential application of hub genes related to fatty acid metabolism as diagnostic markers and therapeutic targets in endometriosis. This approach could improve the diagnosis and treatment of endometriosis, potentially leading to personalized and effective therapeutic interventions. Identifying the key regulatory genes and pathways involved in fatty acid metabolism could provide new insights into the pathogenesis of this disease. *PTGS2, CYP2C9, HSDL2, HSD17B3, ACSL4,* and *CYP2C18* were identified as hub genes in this study. Prostaglandin endoperoxidase synthase 2 (*PTGS2*) encodes COX-2 (13) that is overexpressed in the ectopic endometrium of women with endometriosis compared to that in the normal endometrium of women without the disease (14). *PTGS2* emerged as the central hub gene in the present study, exhibiting the highest score among the identified FAMRDEGs. This gene plays an important role in the inflammatory processes associated with endometriosis, contributing to the progression and physiological manifestation of the disease (15).

Prostaglandin lactone synthase (PTGS2) (16) is a hub gene with significantly upregulated expression in patients with endometriosis, driving prostaglandin synthesis. This process intensifies local inflammatory and pain, a hallmark of endometriosis. PTGS2 activity is closely linked to chronic inflammation, pain perception, and pathological changes, further promoting disease progression. In addition, our study also found that the two cytochrome P450 enzymes (17), namely, CYP2C9 and CYP2C18, play an important role in fatty acid metabolism. Their expression not only influences hormone metabolism but may also disrupt the biological function of the endometrium, highlighting their role in fatty acid metabolism disorders and associated pathophysiological states.

This overexpression was correlated with elevated levels of prostaglandins, which are potent mediators of inflammation and pain. Moreover, *PTGS2* is regulated by various factors, including hormonal therapy, which is a common treatment for endometriosis. Hormone therapy can enhance the expression of *PTGS2*, which may explain why it does not cure this disease (18). In addition, *PTGS2* polymorphisms are linked to an increased risk of endometriosis, with genetic susceptibility mediated by inflammatory pathways (19). Similarly, the upregulation of *HSD17B3* expression may increase the risk of endometriosis (20).

At the same time, acid alcohol esterase 1 (ASAH1) and lipase 2 (HSDL2) play crucial roles in fatty acid hydrolysis and metabolism. Changes in their expression directly impact intracellular energy metabolism, inflammatory response, and tissue remodeling, collectively influencing the biological characteristics and disease progression of endometriosis. The involvement of these central genes in regulating inflammation and fatty acid metabolism provides important clues for our understanding of the complex biology of endometriosis and may become key targets for future research and treatment. Therefore, elucidating their roles in inflammation and tissue remodeling could enhance our understanding of disease mechanisms and provide potential strategies for personalized treatment.

Our study identified several biological pathways closely linked to endometriosis through GO and KEGG enrichment analysis, offering key insights into the disease’s pathogenesis. Pathways associated with fatty acid metabolism, including long-chain fatty acid metabolism and arachidonic acid metabolism, revealed the effects of lipid metabolism on inflammatory responses. The activation of these pathways may elevate intracellular fatty acid levels, thereby intensifying the local inflammatory environment. Notably, fatty acid metabolites such as prostaglandins play a crucial role in modulating immune responses and promoting endometrial growth, potentially exacerbating the pathological status of endometriosis. This observation aligns with previous studies that underscore the fundamental role of arachidonic acid metabolism in the inflammatory processes associated with endometriosis. Jiang et al. (21) demonstrated that arachidonic acid metabolism is the most significantly enriched pathway among the common DEGs identified across various subtypes of endometriosis. This suggests the essential role of this metabolic process in the inflammatory pathogenesis of the disease. Upregulation of the expression of genes involved in the arachidonic acid pathway indicates a heightened inflammatory response, which is a hallmark of endometriosis. In addition, the role of fatty acid metabolism in endometriosis has been corroborated by metabolomic studies. Ortiz et al. (22) reviewed the metabolomic profiles of patients with endometriosis and identified significant alterations in lipid metabolism, including those of fatty acids. These metabolic changes demonstrate the impact of the disease on cellular processes, such as energy production, oxidative stress, and inflammation. The identification of specific metabolites could reveal non-invasive biomarkers for early diagnosis and further elucidate the pathophysiology of endometriosis.

At the same time, the enrichment results indicate that abnormal activity in key signaling pathways is directly linked to cell proliferation and survival, potentially dysregulating the cell cycle, thereby promoting disordered proliferation and migration of endometrial cells and increasing the formation of abnormal endometrial tissue. Meanwhile, signaling pathways associated with apoptosis, such as enrichment of apoptosis signaling pathways, indicate changes in cell survival mechanisms in the disease. The activation of pro-inflammatory cytokines may suppress normal apoptosis, thereby enhancing the survival and persistence of endometrial cells.

In this study, we focused on identifying genetic and molecular factors associated with fatty acid metabolism in endometriosis. However, external influences such as diet, hormone fluctuations, and lifestyle factors are crucial in disease occurrence and progression. These factors may have profound effects on fatty acid metabolism and play a crucial role in the occurrence and development of the disease. Studies have shown that eating habits (23) significantly affect lipid metabolism. A diet rich in omega-3 fatty acids (such as fish and nuts) can reduce chronic inflammation, which is often associated with the pathological processes of endometriosis. By inhibiting the synthesis of inflammatory mediators, omega-3 fatty acids may help alleviate symptoms and slow disease progression.

Antioxidant intake may improve lipid metabolism by reducing oxidative stress, which could influence the risk of developing endometriosis. Hormone levels, especially estrogen and progesterone (24), play a crucial role in regulating fatty acid metabolism. Estrogen can enhance the synthesis and transport of fatty acids by activating genes related to fat metabolism, thereby changing the function of fat cells (hormone fluctuations not only affect metabolism but also affect the expression of identified genes, thereby further interfering with the progress of the disease).

Lifestyle factors such as physical activity and environmental exposure can also affect fatty acid metabolism and disease severity. Lack of exercise is often associated with obesity, which may induce inflammatory responses and aggravate the symptoms of endometriosis. In addition, endocrine disruptors in the environment may affect hormone balance and further change metabolic pathways.

GSEA results indicate upregulation of lipid metabolism and toxicity-related pathways, a finding that has important clinical and therapeutic implications, especially in the management and intervention of endometriosis. Upregulation of lipid metabolism suggests that in a pathological state, the accumulation of fatty acids and related metabolites may lead to intensification of the inflammatory response, which is considered a key factor in promoting the development of endometriosis. Therefore, therapeutic strategies for lipid metabolism may help relieve local inflammation, thereby reducing patient symptoms and improving quality of life.

On the other hand, upregulation of toxic pathways suggests that apoptosis and stress responses may be imbalanced. In endometriosis, ectopic cells may be in a state of persistent stress, resulting in changes in cell physiological function. Recognizing the interaction of these toxic pathways can provide clues to novel therapeutic strategies, such as the use of antioxidants to combat cellular oxidative stress, or the use of small molecules to target specific toxic signaling pathways to restore normal cell function and slow disease progression.

The immune landscape of endometriosis is intricate and encompasses various immune cell types that considerably affect disease pathogenesis. In our study, ssGSEA revealed a correlation between endometriosis and several immune cell types, including activated CD4 + T cells, gamma-delta T cells, CD56 dim natural killer cells, eosinophils, monocytes, natural killer T cells, and plasmacytoid dendritic cells. The peritoneal fluid of women with endometriosis has a higher concentration of activated CD4 + T cells than that of healthy controls, suggesting an altered immune response in these patients. Furthermore, the peritoneal fluid of women with endometriosis displays increased levels of immunosuppressive cytokines, such as IL-10 and IL-12, which may inhibit the activity of activated CD4 + T cells and contribute to immune evasion by endometriotic lesions (25). The presence of these cytokines correlated with a reduction in peritoneal lymphocytes, particularly within the HLA-DR + CD4 + T cell subpopulation, further indicating an impaired immune response (26). In addition, the interactions between T cells and extracellular matrix (ECM) proteins are modified during endometriosis. Activated T cells from women with endometriosis show increased adhesion to ECM proteins, such as collagen IV and fibronectin. This suggests that these interactions might contribute to the pathogenesis of the disease by facilitating the implantation and survival of ectopic endometrial tissue. This enhanced adhesion could be a result of the altered expression of surface antigens on T cells. However, no significant differences in these antigens were observed between patients with endometriosis and healthy controls (27). These results indicate a close correlation between activated CD4 + T cells and endometriosis, which is consistent with our results. In the present study, *ACSL4* had the strongest positive correlation with effector memory CD4 + T cells. In contrast, *HSDL2* showed the strongest negative correlation with activated CD8 + T cells. These results indicate that hub genes (*ACSL4* or *HSDL2*) are promising therapeutic targets for endometriosis.

Endometriosis (EMs) is a disease characterized by chronic inflammation and immune imbalances; a growing number of studies have revealed the key role of immune cells, especially T cell subsets (28), in the occurrence and development of the disease. In this study, we observed a potential correlation of effector memory CD8 + T cells in endometriosis tissues, suggesting that it may be an important factor affecting the immune microenvironment.

Effector memory CD8 + T cells (Tem) are a long-term survival subpopulation of T cells that are highly specific and can quickly recognize and respond to specific antigens. Typically, these cells quickly activate immune responses when the body is re-exposed to the same antigen, enhancing the body’s defense. However, in the context of endometriosis, these cells may be continuously activated by prolonged exposure to antigens in ectopic endometrial tissue. Effector memory CD8 + T cells are able to secrete pro-inflammatory cytokines (29) such as interferon-*γ* (IFN-γ) and tumor necrosis factor-*α* (TNF-α), thereby mediating local inflammatory responses and increasing tissue damage. This inflammatory environment may not only promote the development of ectopic lesions but may also be closely related to the pain symptoms of the disease, as inflammatory mediators can directly or indirectly activate pain-related neural pathways.

Fatty acid synthase 4 (ACSL4) plays a crucial role in fatty acid metabolism and is closely linked to immune cell infiltration in endometriosis, mainly because ACSL4 plays a key role in fatty acid metabolism. ACSL4 influences the polarization of immune cells, especially in macrophages and T cells (30). Specifically, ACSL4 promotes the enhancement of anti-inflammatory response by promoting the synthesis of specific fatty acids (such as arachidonic acid), affecting the polarization process of macrophages, causing them to polarize to M2. This suggests that ACSL4 is not only involved in regulating fatty acid metabolism but also directly affects the function and infiltration patterns of immune cells, making them play a more effective role in the microenvironment of endometriosis. At the same time, ACSL4 further regulates the response of immune cells by changing the lipid composition of the cell membrane (31), which can significantly affect the migration, proliferation, and activation of immune cells, which is directly related to local inflammatory responses. In endometriosis, the expression of ACSL4 may be closely related to the synthesis of pro-inflammatory cytokines, which will lead to increased infiltration of immune cells in ectopic tissues, aggravating local inflammatory responses and corresponding symptoms. Therefore, understanding the specific mechanisms of ACSL4 in immune cell infiltration will help uncover the pathological mechanisms of endometriosis and provide new targets for future therapeutic strategies. Follow-up studies can further explore the improvement of clinical symptoms in patients with endometriosis by regulating ACSL4 expression activities, which will be a potential therapeutic development direction.

Despite our comprehensive analyses, this study has some limitations. First, this study did not combine wet laboratory validation. Rather, it relied only on bioinformatics analyses. Therefore, further experimental validation is needed to confirm our findings. Second, the sample size was small. Therefore, more samples are needed for validation. In addition, there was a lack of clinical validation analyses to ensure that the findings have practical applications.

In our study, key molecular features associated with endometriosis were identified through bioinformatics analysis and computational tools using publicly available gene expression dataset. Specifically, we downloaded endometriosis-related datasets (GSE120103 and GSE25628) from the GEO database, and batch processing was performed using the R package SVA to obtain the integrated gene expression dataset. Subsequently, differential analysis was performed using the R package limma to identify 405 differentially expressed genes. At the same time, six hub genes were screened from the differential genes through the MCC algorithm of the CytoHubba plug-in of the Cytoscape software. These six hub genes present a certain accuracy in the verification model, with an average AUC > 0.7. This suggests that they have high accuracy in the region of patients with endometriosis and healthy individuals. Therefore, our research not only reveals potential biomarkers but also provides a theoretical basis for future clinical applications.

However, owing to time and resource limitations, we have not yet carried out laboratory verification work. We plan to add relevant experiments to future studies to verify the function and mechanism of identified hub genes through *in vitro* and *in vivo* experiments; this will further enhance the application value of our research results and facilitate future research and treatment of endometriosis.

In this study, we aimed to explore the molecular mechanisms of endometriosis by analyzing the GSE120103 and GSE25628 datasets. Endometriosis is a complex gynecological disease with unclear pathogenesis, and so understanding its molecular basis is crucial to developing effective diagnostic and therapeutic methods. Our core analytical steps included identifying and screening differentially expressed genes (DEGs). By comparing the expression profiles of healthy tissues and lesion tissues, a series of genes that were significantly differentially expressed in endometriosis were successfully screened. Subsequently, the CytoHubba plug-in of the Cytoscape software was used along with the MCC algorithm to screen six hub genes from differential genes. These genes have important centrality in the network and likely play a key role in the development of endometriosis.

To verify the clinical application value of these hub genes, we constructed a validation model; the results showed that the average AUC of these genes was >0.7 in patients with endometriosis and healthy individuals, indicating that they have good predictive capabilities and potential biomarker effects.

A larger sample size would better represent genetic diversity and variability in a wider patient population. However, owing to time and resource constraints, we are currently unable to obtain a larger volume of sample data. Future research will consider incorporating more samples in a bid to further explore the complex heterogeneity and potential therapeutic targets of endometriosis. Moreover, we plan to include samples from patients of different ethnicities to more comprehensively explore the heterogeneity of endometriosis.

In conclusion, we systematically integrated and analyzed two GEO datasets and identified 17 DEGs related to fatty acid metabolism using a series of bioinformatics methods. The PPI network identified six hub genes: *PTGS2, CYP2C9, HSDL2, HSD17B3, ACSL4,* and *CYP2C18*. Moreover, ssGSEA immune infiltration analysis revealed that *ACSL4* showed the strongest positive correlation with effector memory CD8 T cells, whereas *HSDL2* showed the strongest negative correlation with activated CD8 T cells. These findings not only enrich our understanding of the molecular mechanisms of the disease but also provide valuable insights for the development of new diagnostic markers and therapeutic targets in the future. We are committed to further experimental validations and clinical application transformations in future studies.

## Data Availability

The original contributions presented in the study are included in the article/Supplementary material, further inquiries can be directed to the corresponding author.

## Footnotes

^1^ https://www.ncbi.nlm.nih.gov/geo/

^2^ http://string-db.org

^3^ https://genemania.org/

^4^ http://rna.sysu.edu.cn/chipbase/

^5^ https://rnasysu.com/encori/
